# Supplementary material for: Humans have a longer period of cortical maturation across depth and hierarchy than macaques
Source: PLoS Biol. 2025 Sep 18;23(9):e3003378. doi: 10.1371/journal.pbio.3003378 (PMC12445471; doi:10.1371/journal.pbio.3003378)
Supplement: S1 Results — Validation of T1w/T2w ratio: MBP Expression in Macaques and R1 mapping in humans. (DOCX) [file pbio.3003378.s001.docx]

## S1 Results

Validation of T1w/T2w ratio: *MBP* Expression in Macaques and R1 Mapping in Humans

To test the robustness of the results obtained by T1w/T2w ratio in both macaques and humans, we compared T1w/T2w ratio with the expression of myelin basic protein (*MBP*) in macaques using single-cell sequence published in Chen et al., 2023^1^. Spatially, *MBP* expression was higher in sensorimotor regions than in association regions (S2A Fig, B *R^2^* = 0.250, *F*(1, 131) = 24.350, *P* < 0.001). The T1w/T2w ratio showed a significant positive correlation with *MBP* expression across cortex (S2C Fig *r* = 0.406, *P* < 0.001), consistent with prior findings^2–8^. For depth-wise validation, *MBP* expression was also significantly higher in deeper layers than in superficial layers (S2D-E Fig, all ANOVAs *F* > 9.236, *P* < 0.001). To assess finer grained correspondence within individual cortical areas, we calculated the correlation coefficient between *MBP* and the T1w/T2w ratio for each CHARM parcel (S2F Fig). Among the 86 parcels that had six histological layers identified in the *MBP* data, 88% (76 parcels) showed a significant correlation (*r* > 0.815, *P* < 0.050). A region-wise permutation test confirmed that this depth-dependent correlation is significantly higher than expected by random chance (*T* = -108.309, *P* < 0.001). These consistent depth-dependent patterns across broad cortical regions and the high proportion of significant correlations at the individual areas level validate the T1w/T2w ratio as a reliable marker for myelin content across both species and provide a robust method for assessing myelination variations across regions and depths.

In humans, we used R1 maps from adult participants aged 18–30 (*N* = 42), provided by Alkemade et al., 2020^9^. Spatially, R1 values were higher in sensorimotor regions compared to association regions (S3A Fig; *R²* = 0.189, *F(1,399)* = 44.4, *P* < 0.001). The T1w/T2w ratio showed a significant positive correlation with R1 across the cortex (S3B Fig, *r* = 0.499, *P* < 0.001), consistent with previous findings. Furthermore, R1 values were significantly higher in deeper cortical layers than in superficial layers (S3C Fig all ANOVAs *P* < 0..001), mirroring the pattern observed in the T1w/T2w ratio.^9^

## Reference

1. Chen, A., Sun, Y., Lei, Y., Li, C., Liao, S., Meng, J., Bai, Y., Liu, Z., Liang, Z., Zhu, Z., et al. (2023). Single-cell spatial transcriptome reveals cell-type organization in the macaque cortex. Cell *186*. https://doi.org/10.1016/j.cell.2023.06.009.

2. Lazari, A., and Lipp, I. (2021). Can MRI measure myelin? Systematic review, qualitative assessment, and meta-analysis of studies validating microstructural imaging with myelin histology. Neuroimage *230*. https://doi.org/10.1016/j.neuroimage.2021.117744.

3. Mancini, M., Karakuzu, A., Cohen-Adad, J., Cercignani, M., Nichols, T.E., and Stikov, N. (2020). An interactive meta-analysis of MRI biomarkers of Myelin. Elife *9*. https://doi.org/10.7554/eLife.61523.

4. van der Weijden, C.W.J., García, D.V., Borra, R.J.H., Thurner, P., Meilof, J.F., van Laar, P.J., Dierckx, R.A.J.O., Gutmann, I.W., and de Vries, E.F.J. (2021). Myelin quantification with MRI: A systematic review of accuracy and reproducibility. Neuroimage *226*. https://doi.org/10.1016/j.neuroimage.2020.117561.

5. Ritchie, J., Pantazatos, S.P., and French, L. (2018). Transcriptomic characterization of MRI contrast with focus on the T1-w/T2-w ratio in the cerebral cortex. Neuroimage *174*. https://doi.org/10.1016/j.neuroimage.2018.03.027.

6. Fulcher, B.D., Murray, J.D., Zerbi, V., and Wang, X.J. (2019). Multimodal gradients across mouse cortex. Proc Natl Acad Sci U S A *116*. https://doi.org/10.1073/pnas.1814144116.

7. Patel, Y., Shin, J., Drakesmith, M., Evans, J., Pausova, Z., and Paus, T. (2020). Virtual histology of multi-modal magnetic resonance imaging of cerebral cortex in young men. Neuroimage *218*. https://doi.org/10.1016/j.neuroimage.2020.116968.

8. Whitaker, K.J., Vendetti, M.S., Wendelken, C., and Bunge, S.A. (2018). Neuroscientific insights into the development of analogical reasoning. Dev Sci *21*. https://doi.org/10.1111/desc.12531.

9. Alkemade, A., Mulder, M.J., Groot, J.M., Isaacs, B.R., van Berendonk, N., Lute, N., Isherwood, S.J., Bazin, P.L., and Forstmann, B.U. (2020). The Amsterdam Ultra-high field adult lifespan database (AHEAD): A freely available multimodal 7 Tesla submillimeter magnetic resonance imaging database. Neuroimage *221*. https://doi.org/10.1016/j.neuroimage.2020.117200.
