# Supplementary figures and images for: Humans have a longer period of cortical maturation across depth and hierarchy than macaques
Source: PLoS Biol. 2025 Sep 18;23(9):e3003378. doi: 10.1371/journal.pbio.3003378 (PMC12445471; doi:10.1371/journal.pbio.3003378)

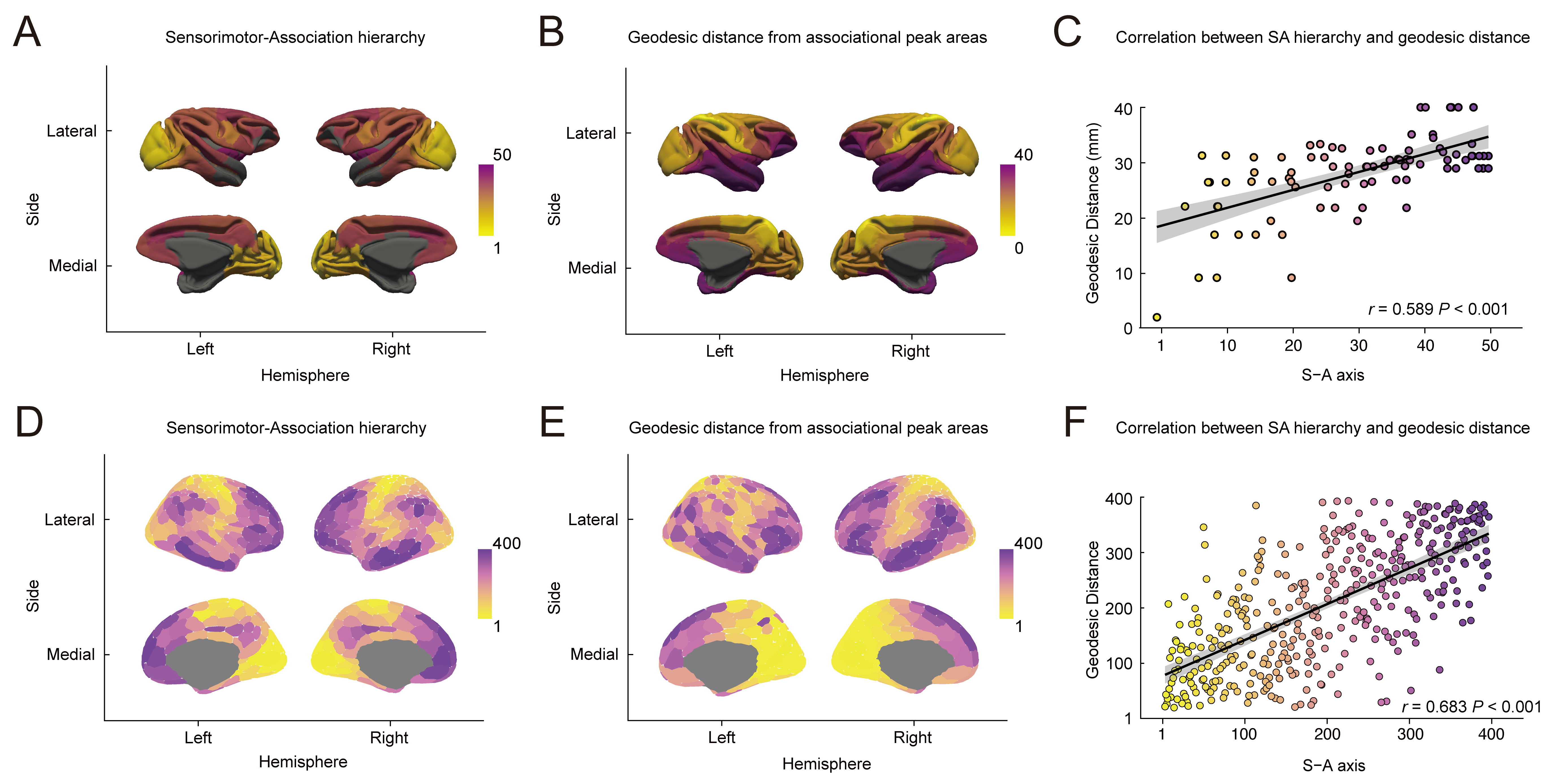

Supplement: S1 Fig — Correlation between geodesic distance and the sensorimotor-association axis. (A, D) Sensorimotor-association axis in macaques (A) and humans (D). (B, E) Geodesic distance from association regions in macaques (B) and humans (E). (C, F) Correlation between S-A axis and geodesic distance in macaques (C, r = 0.589, P < 0.001) and humans (F, r = 0.683, P < 0.001). The data underlying this figure can be found at https://github.com/monami-nishio/prolonged_cortical_maturation. (TIF) [file pbio.3003378.s002.tif]

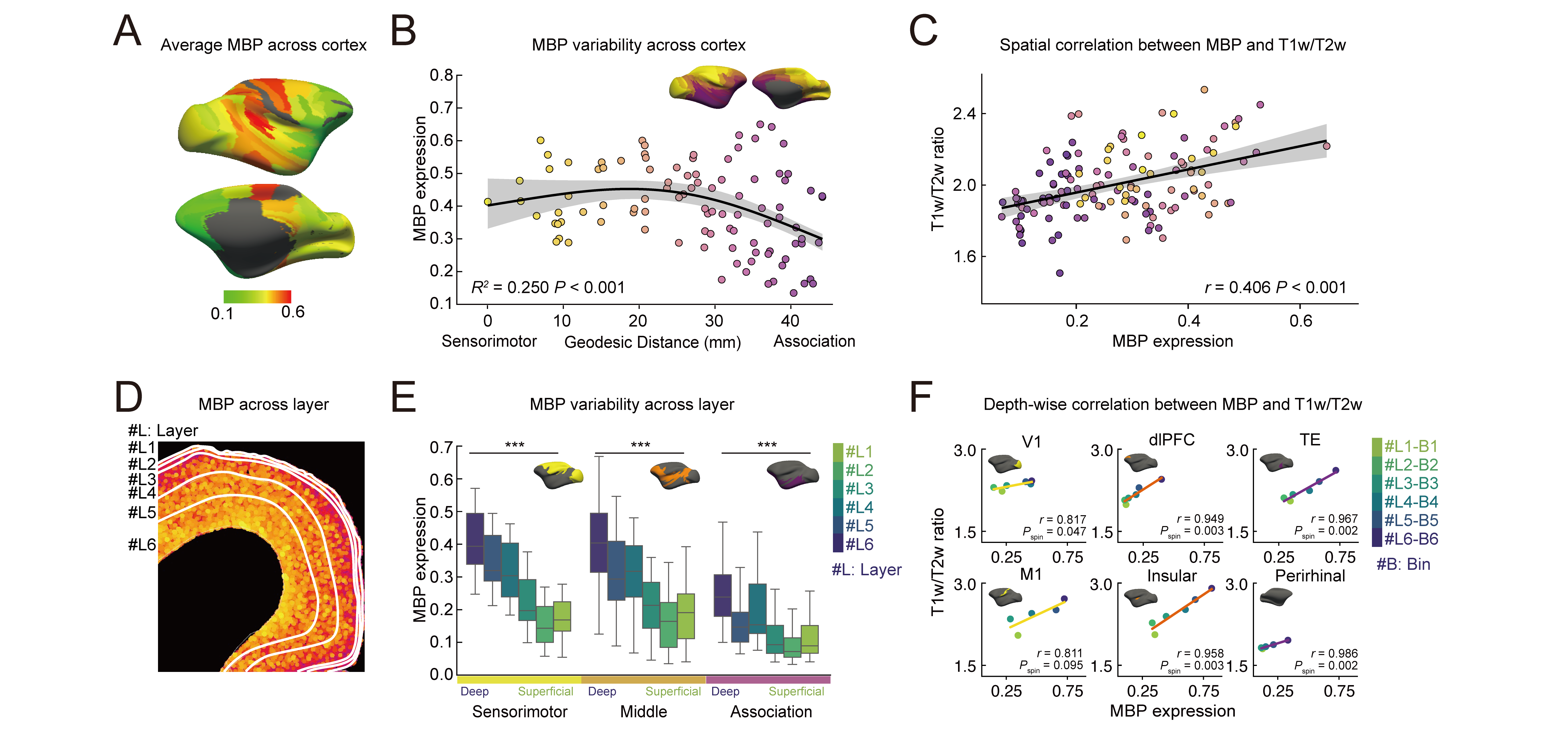

Supplement: S2 Fig — (A) MBP expression across the cortex. (B) MBP expression along the geodesic distance from association regions. Brain regions are arranged on a spectrum from farther (yellow) to closer (purple) to the association centers. The GAM-predicted fit is displayed with a 95% confidence interval (R2 = 0.250, P < 0.001). (C) Correlation between MBP expression and the T1w/T2w ratio across the cortex (r = 0.406, P < 0.001). The regression line is displayed with 95% confidence intervals. (D) Schematic illustration of MBP expression across cortical layers. (E) MBP expression in each layer for sensorimotor (yellow), middle (orange), and association (purple) regions. ANOVA ***P < 0.001. (F) Correlation between MBP expression and T1w/T2w ratio across layers or bins in representative brain regions. The data underlying this figure can be found at https://github.com/monami-nishio/prolonged_cortical_maturation. (TIF) [file pbio.3003378.s003.tif]

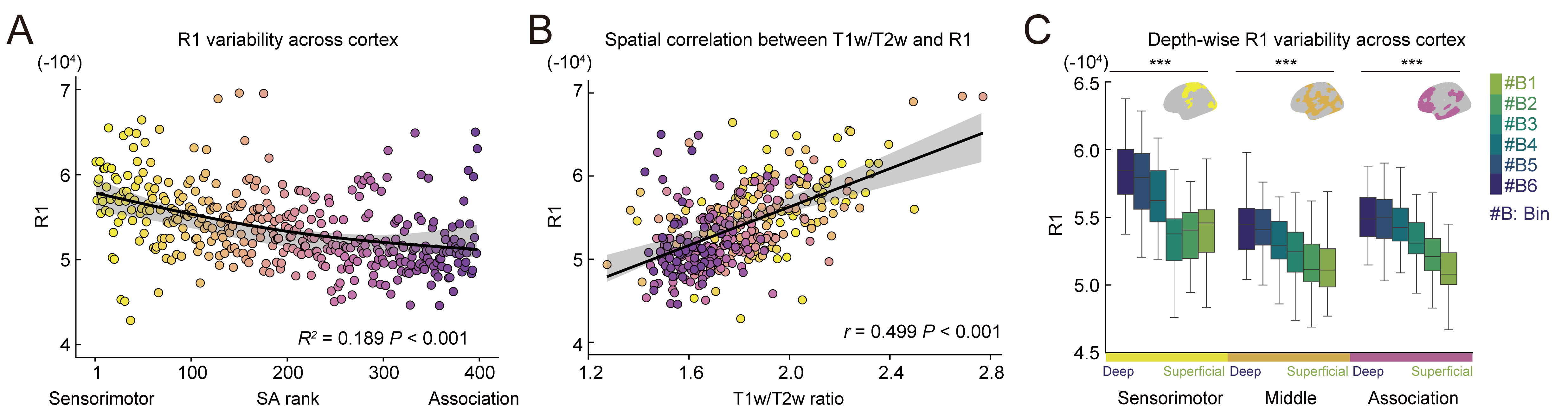

Supplement: S3 Fig — (A) Distribution of R1 along the sensorimotor-association (S-A) axis for humans (R2 = 0.189, P < 0.001). (B) Correlation between T1w/T2w ratio and R1 across the cortex in humans (r = 0.499, P < 0.001). The regression line is displayed with a 95% confidence interval (R2 = 0.209, P < 0.001). (C) Cortical depth-wise R1 within sensorimotor, middle, and association regions in humans (ANOVA ***P < 0.001). The data underlying this figure can be found at https://github.com/monami-nishio/prolonged_cortical_maturation. (TIF) [file pbio.3003378.s004.tif]

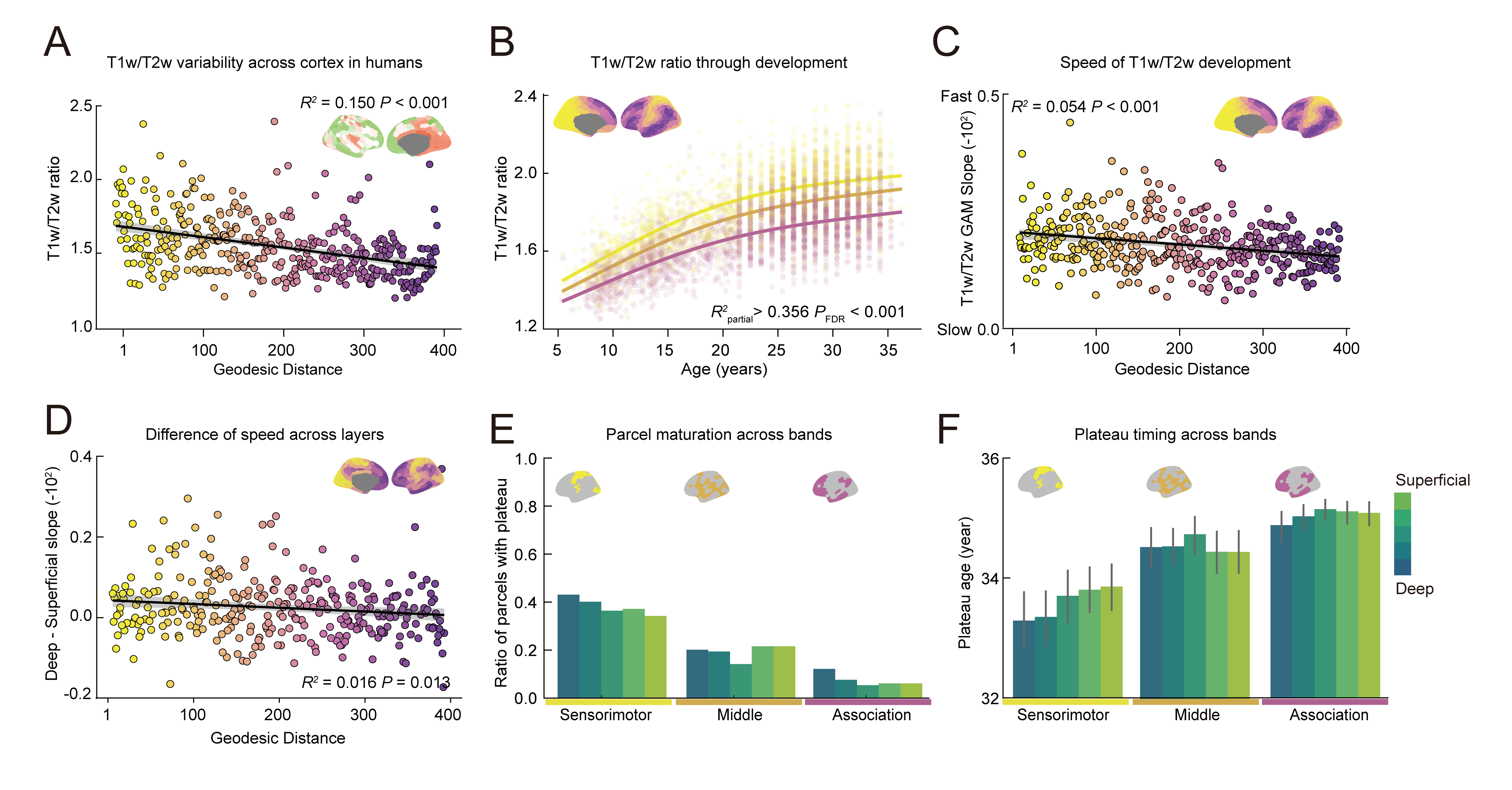

Supplement: S4 Fig — (A) T1w/T2w ratio across the cortex in humans. Brain parcels are aligned along geodesic distance from default mode network regions (R2 = 0.150, P < 0.001). (B) Developmental trajectories of the T1w/T2w ratio for sensorimotor (yellow), middle (orange), and association (purple) cortical areas. Solid lines illustrate Generalized Additive Model (GAM)-predicted fits along with their 95% confidence intervals (Sensorimotor; R2partial = 0.466, PFDR < 0.001, Middle; R2 partial = 0.430, PFDR < 0.001, Association; R2 partial = 0.356, PFDR < 0.001). (C) GAM slope along the geodesic distance for humans. The GAM-predicted fit is displayed with a 95% confidence interval (R2 = 0.054, P < 0.001). (D) Slope differences between the deepest and the most superficial bins along the geodesic distance in humans, with GAM-predicted fits and their 95% confidence intervals (R2 = 0.016, P = 0.013). (E) Proportion of cortical parcels with plateau at each cortical depth bin for sensorimotor, middle, and association regions for humans. (F) Average timing of plateau in each cortical depth bin for sensorimotor, middle, and association regions for humans. The data underlying this figure can be found at https://github.com/monami-nishio/prolonged_cortical_maturation. (TIF) [file pbio.3003378.s005.tif]

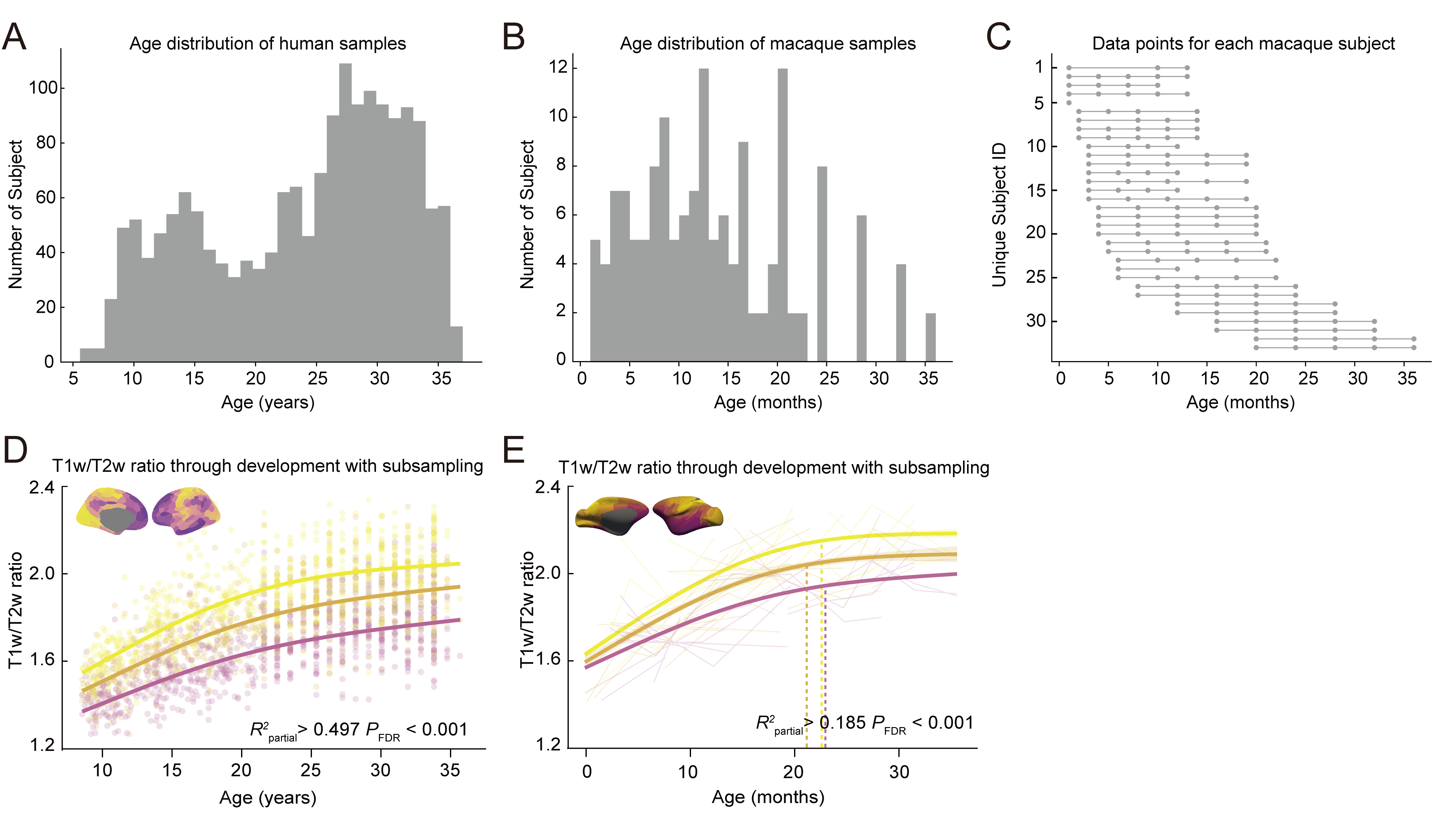

Supplement: S5 Fig — (A) Age distribution of human participants from the HCP-D and HCP datasets. (B, C) Age distribution (B) and scan time points per subject (C) for macaque subjects from the UNC-Wisconsin Rhesus Macaque Neurodevelopment Database. (D, E) Developmental trajectories of the T1w/T2w ratio in sensorimotor (yellow), middle (orange), and association (purple) cortical areas for humans (D) and macaques (E), based on subsampled data. The data underlying this figure can be found at https://github.com/monami-nishio/prolonged_cortical_maturation. (TIF) [file pbio.3003378.s006.tif]
